# Repetitive Transcranial Magnetic Stimulation Improves Handwriting in Parkinson's Disease

**DOI:** 10.1155/2013/751925

**Published:** 2013-05-08

**Authors:** Bubblepreet K. Randhawa, Becky G. Farley, Lara A. Boyd

**Affiliations:** ^1^Graduate Program in Rehabilitation Sciences, University of British Columbia, 212-2177 Wesbrook Mall, Vancouver, BC, Canada V6T 1Z3; ^2^Department of Physiology, University of Arizona, McKale Center 229C, P.O. Box 210096, Tucson, AZ 85721, USA; ^3^Department of Physical Therapy, University of British Columbia, 212-2177 Wesbrook Mall, Vancouver, BC, Canada V6T 1Z3

## Abstract

*Background*. Parkinson disease (PD) is characterized by hypometric movements resulting from loss of dopaminergic neurons in the substantia nigra. PD leads to decreased activation of the supplementary motor area (SMA); the net result of these changes is a poverty of movement. The present study determined the impact of 5 Hz repetitive transcranial magnetic stimulation (rTMS) over the SMA on a fine motor movement, handwriting (writing cursive “l”s), and on cortical excitability, in individuals with PD. *Methods*. In a cross-over design, ten individuals with PD were randomized to receive either 5 Hz or control stimulation over the SMA. Immediately following brain stimulation right handed writing was assessed. *Results*. 5 Hz stimulation increased vertical size of handwriting and diminished axial pressure. In addition, 5 Hz rTMS significantly decreased the threshold for excitability in the primary motor cortex. *Conclusions*. These data suggest that in the short term 5 Hz rTMS benefits functional fine motor task performance, perhaps by altering cortical excitability across a network of brain regions. Further, these data may provide the foundation for a larger investigation of the effects of noninvasive brain stimulation over the SMA in individuals with PD.

## 1. Introduction

Hypometric movements, resulting in diminution of letter size, reduced speed and slow acceleration, typically characterize handwriting in individuals with Parkinson disease (PD) [1–5]. Deficits in handwriting begin with hypometric movements and then may progress to micrographia as PD severity progresses. Specifically, hypometric movements may be related to the impaired ability to maintain adequate muscle force and to process concurrent and forthcoming movement information while writing. Given that individuals with PD suffer from hypometric handwriting [1], we selected this task to consider the effects of noninvasive brain stimulation on hand function. 

Normally, the basal ganglia (BG) play a role in the kinematic scaling of movements [6], but in individuals with PD, suboptimal BG function due to dopamine depletion leads to widespread changes in interconnected brain regions that include decreased activity in the supplementary motor area (SMA) and reduced efferent feedback in the basal ganglia-thalamocortical motor loop [7]. Consequently, individuals with PD show altered activation patterns in the SMA [8–15] and overall less corticocortical excitability [8–10, 12]. Taken together, changes in activation patterns across a broad cortical network and sub-optimal BG function lead to hypometric movements associated with PD. 

Located on medial aspect of the forebrain, the SMA plays a key role in motor selection in sequentially structured tasks such as handwriting. Data from healthy controls suggest that the cortical control of handwriting requires activity in the SMA, motor cortex, and BG in order to produce finely graded precision grip required by handwriting [6]. To generate movements in the absence of external cues, the SMA receives efferent information from the BG and then transmits information that likely helps to prepare movement [16] to M1. However, individuals with PD suffer from overactivation of internal segment of Globus Pallidus internus (GPi) in BG that leads to inhibition of thalamic neurons to cortex, especially to SMA [1–4, 13–15, 17]. Consequently, individuals with PD have decreased activity in SMA owing to diminished efferent feedback from BG-thalamocortical motor loop. Due to poor BG and SMA functions, individuals with PD suffer from a reduced ability to process concurrent and forthcoming information about movement; in the case of handwriting, progressive micrographia results [18].

Repetitive transcranial magnetic stimulation (rTMS) is a non-invasive technique that allows cortical excitability to be altered; when delivered at frequencies ≥5 Hz rTMS cortical excitability may be increased. Past work suggests that rTMS over motor and prefrontal cortex induces a dopamine release in the striatum in people with PD [19–21]. However, this work did not consider whether the application of rTMS might impact functional motor skill performance. Thus, the main aim of the present study was to consider whether the delivery of excitatory rTMS could impact handwriting performance. We targeted the SMA with rTMS based on its known role in handwriting performance [6] in combination with previous reports of altered SMA function in individuals with PD [8, 9]. Past kinematic studies of handwriting [1, 4] suggest it is an ideal task for the study of motor skill function in individuals with PD. 

Given that rTMS over the SMA may shift cortical excitability both locally and in linked cortical areas, we hypothesized that following 5 Hz rTMS individuals with PD would demonstrate improved speed and amplitude of handwriting movements. Tuelings et al. [22] reported that individuals with PD show greater disfluency in writing tasks involving wrist flexion than in the tasks involving wrist extension. Therefore, we predicted that individuals with PD would demonstrate more improvement in the downstroke as compared to the upstroke following 5 Hz rTMS. Finally, given the strong cortico-cortico connections between SMA and M1 we also hypothesized 5 Hz rTMS over SMA would also affect the excitability of M1. To our knowledge, the present study is the first study to assess the effect of rTMS over SMA on a functional motor skill, handwriting, in individuals with PD.

## 2. Materials and Methods

Ten individuals with PD (mean age: 70.5 years) participated (Table 1). To characterize disease status, the motor section of the Unified Parkinson's Disease Rating Scale (UPDRS-III) and Hoehn and Yahr's (H&Y) scores were determined by a physiotherapist prior to the first session of testing while individuals were on medication. Exclusion criteria included (1) age above 80; (2) cognitive dysfunction (i.e., Montreal Cognitive Assessment < 24); (3) history of psychiatric disturbances; (4) any neuromuscular, skeletal, cardiovascular conditions that might interfere in participating in the study; (5) history of seizures/epilepsy, substance abuse or head trauma, stroke, tumor; or (6) severe PD (H&Y stage > 3). Additional exclusion criteria for functional magnetic resonance imaging (fMRI) anatomical scanning and TMS mapping included pacemaker, pregnancy, metallic objects in the body, or claustrophobia. None of the participants presented with significant tremors. 

Participants were tested while on their regular medication schedule; interviews confirmed that medication status did not change during the period of study participation. To control for medication-induced fluctuations in function, all participants were tested at the same time of day for each of the two sessions, two hours before their next medication dosage. That is, the testing was done during second (declining) phase of medication cycle, to capture the maximum add-on effect of rTMS. All participants gave informed, written consent for their participation in the study and all procedures were institutionally and ethically approved. To control variability in participants, all participants were tested using their right hand to write; rTMS was delivered over the left hemisphere for SMA and M1. This study had cross-over design, all participants received 5 Hz and control rTMS over left SMA one week apart. The order of type of stimulation was randomly allocated.

### 2.1. TMS Protocol

**Figure 1:**
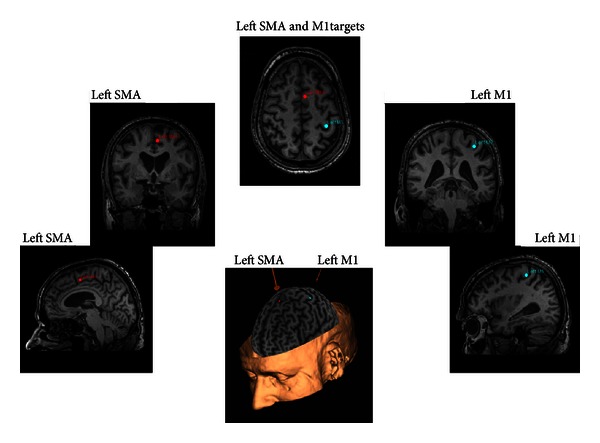
Stereotaxic system for coil placement. Brainsight was used to locate left SMA (as per Talairach coordinates) and left M1; markers were placed to ensure accuracy of coil placement within and across stimulation sessions.

A Magstim Super Rapid stimulator (Magstim Company, Ltd.) was used to deliver the whole TMS, in conjunction with a 70 mm figure-of-eight air-cooled coil. During TMS participants were seated in a semireclined dental chair with arms bent and supported. For the whole stimulation session, over both M1 to determine resting motor threshold (RMT) and SMA for rTMS, the TMS coil was oriented tangentially to the scalp with the handle pointing back and away from midline at 45 degrees. The magnetic stimulus had a biphasic waveform with a pulse width of 400 us. On a separate day, prior to the start of the experiment, each participant had an anatomical MRI scan at the University of British Columbia (UBC) 3T MRI Centre (T1 images TE = 5 ms, TR = 24 ms, 40° flip angle, NEX = 1, thickness = 1.2 mm, FOV = 256 mm). These images were imported into Brainsight TMS neuronavigation software (Rogue Research Inc.) to allow for stereotaxic registration of the participant's brain with the TMS coil for online control of the trajectory of stimulation and to ensure consistency of stimulation location across experimental days. Each participant's brain was transformed into standard Talairach space using Brainsight software. This enabled standardization of rTMS delivery over known Talairach coordinates for the SMA: −5, −3, 52 [23, 24] (Figure 1).

Motor evoked potentials (MEPs) were used to determine the coil position that evoked the maximal response (i.e., the “hot spot”) in the right flexor carpi radialis (FCR). MEP amplitude was monitored by surface electromyography (EMG) over participant's right FCR using the evoked potential unit of the Super Rapid^2^ control unit (Magstim Super Rapid^2^, Magstim Company, Ltd.). Once the location and trajectory of the coil were determined for this hot-spot it was marked using Brainsight to minimize variability. The motor cortical hot-spot was verified at the beginning of each experimental session, as well as before and after rTMS. Following determination of the motor cortical hot spot, RMT was established as the percentage of stimulator output intensity that elicited an MEP > 50 *μ*V in 5 out of 10 trials. To determine the impact of stimulation conditions (5 Hz and control rTMS over SMA) on the excitability of the primary motor cortex we repeated our assessment of RMT on left M1 following 5 Hz stimulation over the SMA. Figure 1 shows the site of stimulation as recorded in Brainsight for left M1 and left SMA.

All participants were naïve to TMS and were blinded to group assignment. 1200 pulses of rTMS stimuli were delivered at 110% of RMT at a frequency of 5 Hz over the SMA [16] (approximately 6 minutes of stimulation). The same protocol was followed for the control, sham stimulation. Control stimulation over SMA was delivered by using an identical custom sham coil that had same look and sound as an active coil but did not induce any current in the underlying cortex. One participant reported scalp discomfort at 110% intensity and thus was stimulated at 100% RMT. All stimulation parameters were in accordance with published safety standards [25]. 

### 2.2. Handwriting

**Figure 2:**
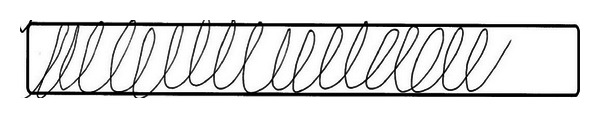
Example of raw data from handwriting task from an individual trial showing a rectangular box of 0.79 by 8 inches; participants were instructed to match the height of their loops to the size of the box.

Handwriting assessments were performed while participants sat at a table adjusted for height to allow the right arm to be comfortably placed with the elbow below the shoulder. Participants were asked to write repetitive cursive loops or “l”s in their everyday style and preferred speed using an ink pen on an 8.5 by 11-inch paper placed on top of a digitizing tablet (WACOM Intuos3 tablet 9X12). The paper contained rectangular boxes of 0.79 by 8 inches and participants were instructed to match the height of their loops to the size of the box (Figure 2). Before starting the experiment, all participants were allowed to practice a trial. Two trials of 15 seconds each were recorded. For each condition (5 Hz versus control rTMS), data was collected twice—prior to rTMS and after rTMS on the same day.

### 2.3. Data Analyses

Kinematic variables of handwriting were quantified using ScriptAlyzer software (NeuroScript, LLC; Tempe, AZ, USA). ScriptAlyzer was used to record position data (*X*-*Y* coordinates) and then calculate the kinematic parameters of interest at a frequency of 200 Hz with a spatial resolution of 0.002 cm. For each loop, the software used the zero velocity crossing to identify two segments, an upstroke and a downstroke. The software automatically eliminated the first loop (up- and downstroke) from each trial. For any trials where freezing or hand repositioning events occurred, the software identified the segment immediately before and after the event and eliminated those segments and the data within that event. After software elimination, stroke segments were visually inspected to verify data. On average, 20 segments were analyzed per subject per test session.

Finally, for each trial, ScriptAlyzer used the averaged segment data to calculate the following kinematic parameters: (1) vertical size (cm); (2) peak vertical velocity (cm/sec); (3) average pen pressure (*z* coordinate). We analyzed parameters of the complete loop as well as separated segments (up- and downstrokes). 

### 2.4. Statistical Analysis

For each of the above dependent variables, a 2 (session: 5 Hz, control rTMS) by 2 (Time: before and after stimulation) ANOVA with repeated measures corrections was performed. The mean of each variable was the dependent measure; SPSS software (v.14) was used for each analysis. Significant interactions (session by time) were decomposed with follow-up *t*-tests. The same statistical test was employed with RMT as the dependent measure to index cortical excitability. Threshold for significance was set to *P* ≤ 0.05.

## 3. Results

### 3.1. Complete Loops

**Figure 3:**
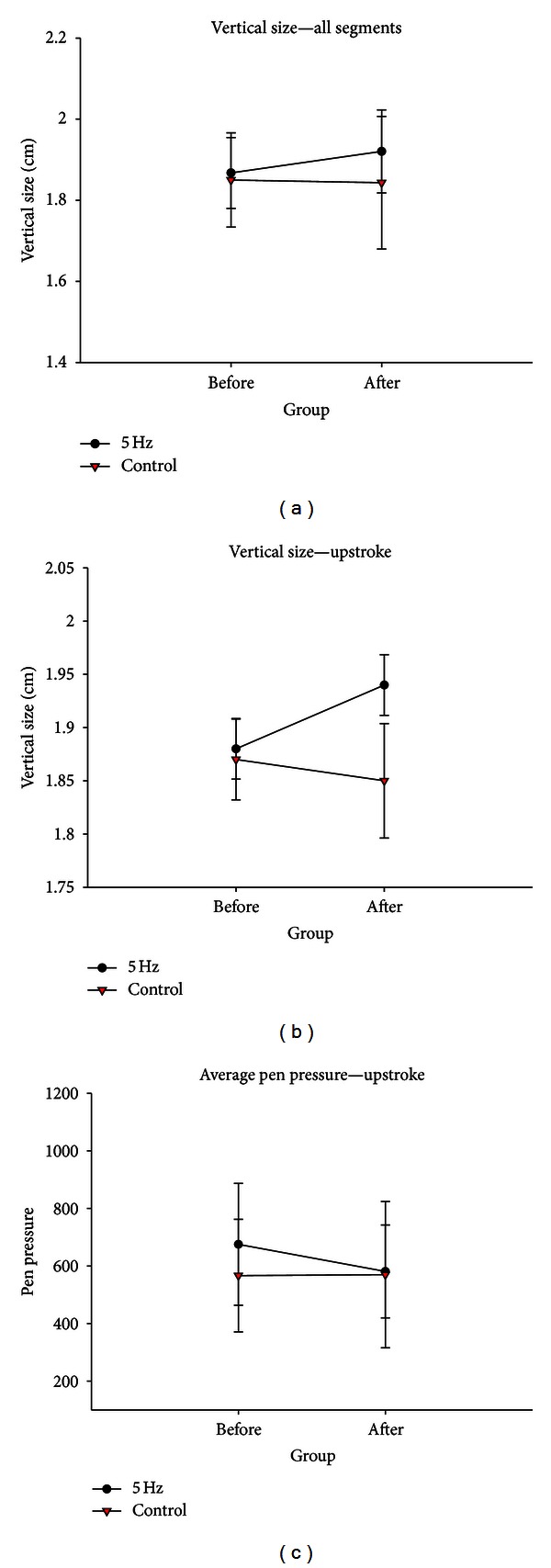
Significant interactions in behavioural data from handwriting task for (a) vertical size, (b) upstroke size, and (c) average pen pressure. Error bars are standard error of the mean (SEM).

(a)* Vertical size*: a significant session by time interaction (*F*(1,9) = 5.59, *P* = 0.04; Table 2; Figure 3(a)) resulted from increased global vertical size for the 5 Hz group at the posttest as compared to control group. 

(b)* Peak vertical velocity*: a significant main effect of time was noted for peak vertical velocity (*F*(1,9) = 10.67, *P* = 0.01). This suggests that both groups wrote faster at the post-test, regardless of stimulation type. Neither the session nor time interaction was significant (Table 2).

(c)* Average pen pressure*: there were no significant effects of rTMS on pen pressure.

### 3.2. Up- and Downstrokes

(a) *Vertical size:* there was significant Session by Time interaction for vertical upstroke size (*F*(1,9) = 9.62, *P* = 0.01). Upstrokes were larger following 5 Hz rTMS (Table 2; Figure 3(b)). No interaction was observed for downstrokes. 

(b) *Peak vertical velocity*: a significant main effect of Time on downstrokes resulted from increased peak vertical velocity (*F*(1,9) = 8.69, *P* = 0.02; Table 2). 

(c) *Average pen pressure*: there was significant Session by Time interaction for upstrokes (*F*(1,9) = 4.93, *P* = 0.05). This was due to the decreased pen pressure following 5 Hz rTMS (Table 2; Figure 3(c)). No interaction was observed for downstrokes.

### 3.3. Motor Cortical Excitability

 A Session by Time interaction was noted for RMT (*F*(1,9) = 5.25, *P* = 0.05). This was the result of lower RMT over M1 following 5 Hz rTMS (*P* = 0.02; Table 3). This was not the case following control rTMS.

## 4. Discussion

 This is the first study to demonstrate short-term changes in functional fine motor task performance following 5 Hz rTMS over SMA in individuals with PD. Specifically, we noted that 5 Hz rTMS over SMA increased the global size of handwriting as shown by larger “l”s, specifically upstroke height increased and pen pressure decreased following rTMS over SMA. Van Gemmert et al. [26] reported that individuals with PD undershoot when asked to match their letter height to a target box. In the present study, we show that 5 Hz rTMS countered the undershooting of letter height, at least in the short term. We speculate that 5 Hz rTMS over SMA may alter corticostriatal and corticocortical connectivity perhaps by exciting an otherwise hypoactive SMA and its projections to the BG, M1, and other motor areas. The BG play an important role in motor behavior. According to a hypothetical model, the putamen controls GPi, both directly and indirectly. In PD, the balance between putamen and GPi and globus pallidus-externus (GPe) is altered due to loss of dopamine. The SMA acts with the basal ganglia reciprocally to prepare movements [27–31], forming a corticosubcortical loop. The SMA also sends information to M1 for final output [17]. Our data suggest that 5 Hz rTMS over SMA helped to compensate for corticostriatal imbalance by imposing an efferent influence on BG output and enhanced cortico-cortical connections, thus enabling participants to generate a larger vertical letters following stimulation.

 5 Hz rTMS also led to decrease in the amount of pen tip pressure, during the now larger upstroke. Other work suggests that PD leads to an inefficient recruitment of muscle force, deficits in amplitude and/or velocity scaling, and rigidity in muscle groups [7, 32], resulting in jerky movements [22]. The M1 is well known to encode the force requirements of movement [33]. Importantly, we noted lower motor thresholds for M1 following 5 Hz rTMS over the SMA [34, 35]. Given the strong cortico-cortico linkages between the SMA and M1 this result is not surprising. Taken together, the decrease in pen pressure and reduction in the threshold for stimulation of M1 following 5 Hz rTMS suggest that non-invasive brain stimulation facilitated the cortical control of force perhaps by moving M1 towards a more excitable state. To better understand the mechanism(s) of these improvements, future studies should attempt to quantify dopamine release in different areas of the brain, but especially in the BG, after 5 Hz rTMS over SMA in individuals with PD. 

In addition, we noted that participants improved significantly in writing size for upstrokes as compared to downstrokes after 5 Hz rTMS over SMA. This is contrary to our hypothesis and may be attributed to the fact that individuals with PD have more tonic activation of flexor muscles and reduced control of wrist flexion [22]. Writing curved loops involve finger and wrist extension for upstrokes and finger/wrist flexion for downstrokes. Therefore, rTMS may have facilitated the easier movement of wrist extension (required by upstrokes). Alternatively, it is possible that faster downstrokes resulted from the larger amplitude of movement upward. Future studies should endeavor to use of EMG over the wrist flexors and extensors to directly assess the impact of 5 Hz rTMS over SMA on muscle activity. However, our handwriting data do indicate that participants were able to generate larger letters at their preferred speed following 5 Hz rTMS over SMA as compared to after control rTMS. It is possible that improved handwriting was attributable to better overall muscular control following stimulation. 

We also revealed main effects of time for peak vertical velocity. Since both groups improved, the possibility of a placebo effect cannot be excluded [20]. In fact, placebo effects have been noted to induce an endogenous dopamine release in the BG [36]. However, it is also possible that these effects were attributable to a practice effect. Given that each individual in the present study was in the declining phase of his or her medication cycle, demonstration of improved performance associated with a repetition of the handwriting task is not trivial. The possibility that skilled motor practice improved handwriting provides support for future motor learning and rehabilitation trials of this function in people with PD. 

A limitation of the present study was that all participants were stimulated on medication. However, we did control for medication cycle effects by testing individuals in the same relative phase two hours before their next medication dosage for each session. Secondly, small sample size may have limited our findings. To control for variability in the sample we employed a cross-over design and all participants were both right handed and tested using their right hand. All participants were naïve to rTMS. In addition, none of the past work has assessed the impact of rTMS on motor function, therefore, it was difficult to calculate adequate sample size and run corrections for multicomparisons. Thirdly, we applied rTMS over Talairach coordinates for SMA, which may have altered our ability to impact both flexion and extension of wrist. Future studies may aim for more disperse delivery of rTMS for more global effects. Other methodological limitations may include the heterogeneity of participants in terms of age and gender bias. 

## 5. Conclusions

Taken together, results of the present study reveal that 5 Hz rTMS over the SMA can influence several key aspects of handwriting including vertical size and axial pressure in individuals with PD, at least in the short term. Although the current study cannot elucidate the exact mechanism by which 5 Hz rTMS induced these effects, our data suggest that brain stimulation over SMA altered excitability within the BG-SMA-M1 loop, which led to greater M1 excitability and improved handwriting function. The data reported here represent a first step in determining the potential therapeutic utility of rTMS over the SMA in individuals with PD. In future, rTMS can be combined with other therapeutic modalities for rehabilitation in individuals with PD. Given our promising early results, future work should attempt to elucidate the mechanism(s) associated with the changes in function reported here and examine the cumulative impact of repeated sessions of 5 Hz rTMS over SMA on motor function in individuals with PD.

## Figures and Tables

**Table 1:** Participant Characteristics.

P	Sex	Age	MoCA	UPDRS-III	H&Y	DH	More affected side	Disease duration (years)	Medication (daily dose—mg)
1	M	52	25	9	1.5	R	L	4	Levodopa/Carbidopa 100/25 QID Entacapone 200 QID Pramipexole 1 QID
2	M	73	28	18	1.5	R	R	10	Levodopa/Carbidopa CR 200/50 QID Levodopa/Carbidopa/Entacapone 100/25/200 QID Levodopa/Carbidopa 120/25 TID
3	M	77	27	11	1.5	R	L	4	Levodopa/Carbidopa 250 QD
4	M	77	24	13	1.5	R	R	4	Levodopa/Carbidopa CR 100/25 QID Levodopa/Carbidopa 100/25 BID
5	M	71	29	7	1	R	R	8	Levodopa/Carbidopa CR 200-50 TID Rasagiline 1 QD
6	M	77	29	9	1.5	R	L (more axial)	3	Levodopa/Carbidopa CR 100/25 QID
7	M	72	26	7	1.5	R	R (more axial)	5	Levodopa/Carbidopa 200/50 q5H Pramipexole 1 TID
8	F	64	28	5	1.5	R	R (more axial)	6	Levodopa/Carbidopa CR 100/25 q5H Pramipexole 1 TID Rasagiline 0.5 QD
9	M	64	30	6	1.5	R	L	4	Levodopa/Carbidopa 100/25 QID Pramipexole 0.5 QID
10	M	78	29	9	1.5	R	R	3	Levodopa/Carbidopa CR 200/50 QID

P: participant, Age: years, F: female, M: male, R: right; L: Left, MoCA: Montreal Cognitive Assessment, UPDRS-III: Unified Parkinson's Disease Rating Scale-motor section, H&Y: Hoehn and Yahr's stages, DH: dominant hand, CR: controlled release, QD: one/day, BID: two times/day, TID: three/day, QID: Four/day, q5H: Five/day.

**Table 2:** Average means (SD) before and after rTMS for each group and *P* values for all segments, upstrokes and downstrokes.

Variable	All segments			Upstroke			Downstroke		
	5 Hz	Control	*P* value	5 Hz	Control	*P* value	5 Hz	Control	*P* value
Vertical size (cm)									
Before	1.87 (0.09)	1.85 (0.12)	0.04^∗2^	1.88 (0.09)	1.87 (0.12)	0.01^∗2^	1.86 (0.09)	1.84 (0.12)	NS
After	1.92 (0.10)	1.84 (0.16)		1.94 (0.09)	1.85 (0.17)		1.90 (0.12)	1.84 (0.16)	
Peak Vertical velocity (cm/sec)									
Before	6.97 (1.72)	7.54 (1.08)	0.01^∗1^	7.41 (1.48)	8.11 (1.31)	NS	6.55 (2.07)	7.06 (1.38)	0.02^∗1^
After	7.99 (1.90)	7.98 (1.62)		8.34 (1.89)	8.48 (1.62)		7.72 (2.06)	7.59 (1.86)	
Average pen Beforessure									
Before	658.94 (209.10)	546.43 (204.47)	NS	675.30 (211.95)	566.43 (195.68)	0.05^∗2^	642.39 (211.93)	536.50 (207.25)	NS
After	564.64 (154.47)	550.07 (254.76)		581.09 (161.34)	570.02 (254.05)		543.31 (150.10)	528.36 (259.84)	

NS: not significant; ^∗1^significant main effect of time; ^∗2^significant session by time interaction.

**Table 3:** Average means (SD) before and after rTMS for each group, and *P* values for RMT.

Variable	TMS		*P* value
	Active	Sham	
RMT			
Before	63.1 (10.55)	62.3 (8.08)	0.05*
After	60.2 (9.14)	61.2 (8.59)	

*Significant SESSION by TIME interaction.

## References

[1] Teulings HL, Stelmach GE (1991). Control of stroke size, peak acceleration, and stroke duration in Parkinsonian handwriting. *Human Movement Science*.

[2] Flash T, Henis E, Inzelberg R, Korczyn AD (1992). Timing and sequencing of human arm trajectories: normal and abnormal motor behaviour. *Human Movement Science*.

[3] Longstaff MG, Mahant PR, Stacy MA, Van Gemmert AWA, Leis BC, Stelmach GE (2003). Discrete and dynamic scaling of the size of continuous graphic movements of parkinsonian patients and elderly controls. *Journal of Neurology Neurosurgery and Psychiatry*.

[4] Van Gemmert AWA, Teulings HL, Stelmach GE (2001). Parkinsonian patients reduce their stroke size with increased processing demands. *Brain and Cognition*.

[5] Tucha O, Mecklinger L, Thome J (2006). Kinematic analysis of dopaminergic effects on skilled handwriting movements in Parkinson’s disease. *Journal of Neural Transmission*.

[6] Desmurget M, Grafton ST, Vindras P, Gréa H, Turner RS (2004). The basal ganglia network mediates the planning of movement amplitude. *European Journal of Neuroscience*.

[7] Contreras-Vidal JL, Stelmach GE (1995). A neural model of basal ganglia-thalamocortical relations in normal and parkinsonian movement. *Biological Cybernetics*.

[8] Eckert T, Peschel T, Heinze HJ, Rotte M (2006). Increased pre-SMA activation in early PD patients during simple self-initiated hand movements. *Journal of Neurology*.

[9] Buhmann C, Glauche V, Stürenburg HJ, Oechsner M, Weiller C, Büchel C (2003). Pharmacologically modulated fMRI—cortical responsiveness to levodopa in drug-naive hemiparkinsonian patients. *Brain*.

[10] Ceballos-Baumann AO, Boecker H, Bartenstein P (1999). A positron emission tomographic study of subthalamic nucleus stimulation in Parkinson disease: enhanced movement-related activity of motor-association cortex and decreased motor cortex resting activity. *Archives of Neurology*.

[11] Haslinger B, Erhard P, Kämpfe N (2001). Event-related functional magnetic resonance imaging in Parkinson’s disease before and after levodopa. *Brain*.

[12] Jahanshahi M, Jenkins IH, Brown RG, Marsden CD, Passingham RE, Brooks DJ (1995). Self-initiated versus externally triggered movements—I. An investigation using measurement of regional cerebral blood flow with PET and movement-related potentials in normal and Parkinson’s disease subjects. *Brain*.

[13] Jenkins IH, Fernandez W, Playford ED (1992). Impaired activation of the supplementary motor area in Parkinson’s disease is reversed when akinesia is treated with apomorphine. *Annals of Neurology*.

[14] Playford ED, Jenkins IH, Passingham RE, Nutt J, Frackowiak RSJ, Brooks DJ (1992). Impaired mesial frontal and putamen activation in Parkinson’s disease: a positron emission tomography study. *Annals of Neurology*.

[15] Rascol O, Sabatini U, Chollet F (1994). Normal activation of the supplementary motor area in patients with Parkinson’s disease undergoing long-term treatment with levodopa. *Journal of Neurology Neurosurgery and Psychiatry*.

[16] Hamada M, Ugawa Y, Tsuji S (2008). High-frequency rTMS over the supplementary motor area for treatment of Parkinson’s disease. *Movement Disorders*.

[17] Tanji J, Taniguchi K, Saga T (1980). Supplementary motor area: neuronal response to motor instructions. *Journal of Neurophysiology*.

[18] Van Gemmert AWA, Teulings HL, Contreras-Vidal JL, Stelmach GE (1999). Parkinson’s disease and the control of size and speed in handwriting. *Neuropsychologia*.

[19] Strafella AP, Paus T, Barrett J, Dagher A (2001). Repetitive transcranial magnetic stimulation of the human prefrontal cortex induces dopamine release in the caudate nucleus. *The Journal of Neuroscience*.

[20] Strafella AP, Paus T, Fraraccio M, Dagher A (2003). Striatal dopamine release induced by repetitive transcranial magnetic stimulation of the human motor cortex. *Brain*.

[21] Ji YK, Eun JC, Won YL (2008). Therapeutic effect of repetitive transcranial magnetic stimulation in Parkinson’s disease: analysis of [^11^C] raclopride PET study. *Movement Disorders*.

[22] Teulings HL, Contreras-Vidal JL, Stelmach GE, Adler CH (1997). Parkinsonism reduces coordination of fingers, wrist, and arm in fine motor control. *Experimental Neurology*.

[23] Picard N, Strick PL (1996). Motor areas of the medial wall: a review of their location and functional activation. *Cerebral Cortex*.

[24] Colebatch JG, Deiber MP, Passingham RE, Friston KJ, Frackowiak RSJ (1991). Regional cerebral blood flow during voluntary arm and hand movements in human subjects. *Journal of Neurophysiology*.

[25] Wassermann EM (1998). Risk and safety of repetitive transcranial magnetic stimulation: report and suggested guidelines from the International Workshop on the Safety of Repetitive Transcranial Magnetic Stimulation, June 5–7, 1996. *Electroencephalography and Clinical Neurophysiology*.

[26] Van Gemmert AWA, Adler CH, Stelmach GE (2003). Parkinson’s disease patients undershoot target size in handwriting and similar tasks. *Journal of Neurology, Neurosurgery and Psychiatry*.

[27] Evarts EV, Wise SP (1984). Basal ganglia outputs and motor control. *Ciba Foundation Symposium*.

[28] Martin KE, Phillips JG, Iansek R, Bradshaw JL (1994). Inaccuracy and instability of sequential movements in Parkinson’s disease. *Experimental Brain Research*.

[29] Nolte J (1999). *The Human Brain: An Introduction to Its Functional Anatomy*.

[30] Romo R, Schultz W (1992). Role of primate basal ganglia and frontal cortex in the internal generation of movements. III. Neuronal activity in the supplementary motor area. *Experimental Brain Research*.

[31] Elahi B, Elahi B, Chen R (2009). Effect of transcranial magnetic stimulation on parkinson motor function—systematic review of controlled clinical trials. *Movement Disorders*.

[32] Berardelli A, Rothwell JC, Thompson PD, Hallett M (2001). Pathophysiology of bradykinesia in parkinson’s disease. *Brain*.

[33] Georgopoulos AP, Ashe J, Smyrnis N, Taira M (1992). The motor cortex and the coding of force. *Science*.

[34] Pascual-Leone A, Valls-Sole J, Wassermann EM, Hallett M (1994). Responses to rapid-rate transcranial magnetic stimulation of the human motor cortex. *Brain*.

[35] Pascual-Leone A, Valls-Solé J, Brasil-Neto JP, Cammarota A, Grafman J, Hallett M (1994). Akinesia in Parkinson’s disease. II. Effects of subthreshold repetitive transcranial motor cortex stimulation. *Neurology*.

[36] Strafella AP, Ko JH, Monchi O (2006). Therapeutic application of transcranial magnetic stimulation in Parkinson’s disease: the contribution of expectation. *NeuroImage*.

